# Assessment of *Theileria equi* and *Babesia caballi* infections in equine populations in Egypt by molecular, serological and hematological approaches

**DOI:** 10.1186/s13071-016-1539-9

**Published:** 2016-05-04

**Authors:** Mona S. Mahmoud, Nadia T. Abu El-Ezz, Sobhy Abdel-Shafy, Somia A. Nassar, Amira H. El Namaky, Wagdy K. B. Khalil, Don Knowles, Lowell Kappmeyer, Marta G. Silva, Carlos E. Suarez

**Affiliations:** Parasitology and Animal Diseases Department, National Research Center, 33 Bohouth St., Dokki, 12622 Giza, Egypt; Cell Biology Department, National Research Center, 33 Bohouth St., Dokki, 12622 Giza, Egypt; Animal Disease Research Unit, Agricultural Research Service, USDA, WSU, Pullman, WA USA; Department of Veterinary Microbiology and Pathology, Washington State University, Pullman, WA USA

**Keywords:** *Babesia*, *Theileria*, Equine, Nested PCR, Competitive ELISA, IFAT, Hemogram

## Abstract

**Background:**

Equine piroplasmosis (EP) caused by *Theileria equi*, *Babesia caballi*, or both, contributes to significant economic loss in the equine industry and remains uncontrolled in Egypt. This study focuses on surveying *T. equi* and *B. caballi* infections and hematological disorders in equine populations in Egypt.

**Methods:**

*Theileria equi* and *B. caballi* infections were assessed in blood from 88 horses and 51 donkeys in Egypt using light microscopy, indirect immunofluorescent antibody test (IFAT), nested PCR (nPCR), and competitive-ELISA (cELISA) assays. PCR products were examined for specificity by DNA sequencing. Hematological alterations were evaluated using a standard cell counter.

**Results:**

Microscopic analysis revealed EP infection in 11.4 % and 17.8 % of horses and donkeys respectively. IFAT detected 23.9 % and 17.0 % infection of *T. equi* and *B. caballi*, respectively, in horses, and 31.4 % of *T. equi* and *B. caballi* in donkeys. *T. equi* cELISA detected 14.8 % and 23.5 % positive horses and donkeys, respectively, but the *B. caballi* RAP-1-based cELISA failed to detect any positives, a result hypothesized to be caused by sequence polymorphism found in the *rap-1* genes. Nested-PCR analysis identified 36.4 % and 43.1 % positive horses and donkeys, respectively for *T. equi* and it also identified 19.3 % and 15.7 % positive horses and donkeys, respectively for *B. caballi*. The overall EP incidence found in the population under study was relatively high and comparable regardless of the diagnostic method used (56.8 % using nPCR and 48.9 % using IFAT). Hematologic analysis revealed macrocytic hypochromic anemia and thrombocytopenia in all piroplasma-infected horses.

**Conclusions:**

The data confirm relatively high levels of EP, likely causing hematological abnormalities in equines in Egypt, and also suggest the need for an improved serological test to diagnose *B. caballi* infection in this region.

**Electronic supplementary material:**

The online version of this article (doi:10.1186/s13071-016-1539-9) contains supplementary material, which is available to authorized users.

## Background

Equine piroplasmosis (EP), an infectious tick-borne disease of the Equidae, is caused by two intra-erythrocytic hemoprotozoans, *Theileria equi* (formerly *Babesia equi*) and *Babesia caballi*, in most tropical and subtropical areas, and some temperate zones of the world [1, 2]. The disease can cause significant economic losses in the equine industry globally. *Theileria equi* is considered a more virulent species than *B. caballi,* [3–5]. However, they share the same competent tick vectors, including *Hyalomma excavatum* [6] and mixed *T. equi* and *B. caballi* infections are common in endemic areas [7]. Both *B. caballi* and *T. equi* are responsible for hemolytic disease which is characterized by fever, anemia, red urine, jaundice, edema, drop in body mass and even death [8, 9]. Clinical signs of equine piroplasmosis are often non-specific, precluding accurate diagnosis [10]. Currently, specific diagnosis can be obtained by microscopic examination, indirect immunofluorescent antibody test (IFAT), enzyme-linked immunoassays (ELISA) and polymerase chain reaction (PCR). Sensitive and specific tests for EP are required for disease control and preventing introduction of parasites into countries that are regarded free of infection/disease.

Two competitive-ELISAs (cELISA) were developed for the detection of antibody to *T. equi* and *B. caballi*. The *T. equi* cELISA uses monoclonal antibody (mAb 36/133.97) to *T. equi* merozoite antigen (EMA)-1, while the *B. caballi*-specific cELISA is based on a monoclonal antibody to Bc48 (79/17.18.5), reactive with a member of the *Babesia* sp. rhoptry-associated protein (RAP)-1 family. Recent data suggests that the RAP-1 based cELISA lacks sensitivity for the detection of *B. caballi* - infected equids in South Africa and Israel, and the loss of sensitivity is hypothesized to be due to *rap-1* sequence variations among strains [11, 12].

PCR-based detection of parasites has been reported to have higher sensitivity and specificity compared with serological assays, but these tests are not routinely performed as a diagnostic tool [13–18]. Detection of parasite DNA by PCR is also useful for the early detection of acute phase of infection, when antibodies are not yet detectable. Yet, serological and molecular methods are more reliable than microscopy for detecting persistent infections with low levels of parasitemia. For example, the ability of nested PCR (nPCR) to diagnose sub-clinical infections could help prevent exportation of infected animals as well as in determining the efficiency of pharmacological treatments [18].

Previous data collected in Egypt, suggest high prevalence of EP caused by *T. equi*, which is consistent with the current lack of control measures [19, 20]. Furthermore, no studies on infection of equids with *B. caballi* were previously reported, and surveys using sensitive and specific state-of-the-art diagnostic techniques are required to assess the overall impact of EP on the equine populations in Egypt. Importantly, the possible association between infection with the parasites causing EP, and the occurrence of hematological disorders in equine populations in Egypt also remains an important knowledge gap [20].

Using state-of-the-art diagnostic methods, this study provides an epidemiological snapshot of infection of *B. caballi* and *T. equi* infections in horses and donkeys in selected locations in Egypt, explores the association of hematological parameters with infection status and provides rationale for the selection of diagnostic tests depending on need. Detection of infection was correlated with occurrence of hematological changes suggesting possible long term health impact on infected equids.

## Methods

### Animals and ethical approval

A total of 88 horses from Cairo and 51 donkeys from the Giza zoological garden were investigated for infection with *T. equi* and/or *B. caballi*. The protocol used in these animal studies and all animal handling was approved by the Egyptian Medical Research Ethics Committee of NRC (No. 14–126).

### Blood specimens, microscopic detection, and immunofluorescent assay

Whole blood was collected from animals by jugular vein puncture using Vacutainer tubes® with or without anti-coagulant. Blood with EDTA were used for preparation of blood films, for hematological evaluation and for DNA extractions using FTA® Elute cards (Whatman Cat. No. WB120410). Blood smears were stained with Giemsa stain for 15 min and examined by analyzing twenty five fields in each thin blood film at a 1000× magnification [21]. The indirect immunofluorescent slides of *T. equi* and *B. caballi,* as well as the control equine sera were kindly donated by VMRD Inc. (Pullman, WA, USA). The IFAT was performed using equine sera at a 1:50 dilution.

### Competitive-inhibition ELISA

The cELISA *T. equi* and *B. caballi* test kits used in this study were kindly provided by VMRD Inc. (Pullman, WA, USA). Assays were performed following the manufacturer’s instructions. Optical density (O.D.) values were determined at 650 nm using ELX 800 universal microplate reader Bio-TEK instruments, INC, USA. The results were expressed as a value of the percent inhibition (%I) according to the following formula:

$$ \left(\%I\right):\ \%I = 100\mathit{\hbox{-}}\left[\left( sample\ O.D.\times 100\right)\ /\left( mean\  negative\  control\ O.D.\right)\right]. $$

The manufacturer of the cELISA kit established the %I value of 40 as the cut-off [1, 22]. Thus samples with %I above 40 are considered as positive, and below 40, considered as negative.

### Nested PCR

Total genomic DNA (gDNA) was extracted from whole blood using FTA® Elute cards following the manufacturer’s instructions. The PCRs were performed using 5–10 μl eluted DNA in a final volume of 25 μl containing 12.5 μl JumpStart RED Taq Ready Mix PCR reaction mix (Sigma-Aldrich), and 10 pmol of each primer. The oligonucleotide primer sequences are shown in Table 1. The thermocycling conditions used for either *T. equi* and *B. caballi* amplification were: 95 °C for 3 min, followed by 25 cycles, consisting of denaturation at 95 °C for 15 s in external reaction and 5 s in nested reaction, annealing at 60 °C for 15 s in external reaction and 5 s in nested reaction and extension at 72 °C for 15 s in external reaction and 5 s in nested reaction. A final extension cycle at 72 °C for 5 min was performed and reactions were cooled to 15 °C.

**Table 1:** Oligonucleotide primer pairs used in PCR amplifications for the detection of *Theileria equi* and *Babesia caballi* in equines

Parasite	Primer name	Gene name	PCR reaction	Amplicon size	Primer sequence	Reference
*T. equi*	Beq-F	*ema-1*	External	567 bp	5'-GAG GAG GAG AAA CCC AAG-3'	Baptista et al [36]
	Beq-R				5'-GCC ATC GCC CTT GTA GAG-3'	
	BeqN-F		Nested	229 bp	5'-TCA AGG ACA ACA AGC CAT AC-3'	
	BeqN-R				5'-TTG CCT GGA GCC TTG AAG-3'	
*B. caballi*	Bca-F	*rap-1*	External	375 bp	5'-GATTACTTGTCGGCTGTGTCT-3'	Schwint et al [37]
	Bca-R				5'-CGCAAGTTCTCAATGTCAG-3'	
	BcaN-F		Nested	224 bp	5'-GCTAAGTACCAACCGCTGA-3'	
	BcaN-R				5'-CGCAAGTTCTCAATGTCAG-3'	

The primer sets used for the primary reaction were: Beq-F and Beq-R for the amplification of the *ema-1 T. equi* gene, and Bca-F and Bca-R for the amplification of the *rap-1 B. caballi* gene. The primer sets used for nested PCR reaction were: BeqN-F and BeqN-R for the amplification of the *ema-1 T. equi* gene, and BcaN-F and BcaN-R for the amplification of the *rap-1 B. caballi* gene

*Theileria equi* and *B. caballi* gDNA derived from cultured Florida *T. equi* and Puerto Rico *B. caballi* strains, respectively, used as positive controls were obtained from the OIE Equine piroplasmosis reference laboratory, Animal Disease Research Unit, Agricultural Research Service, USDA, WSU, Pullman, WA. A negative control with no DNA template was included for PCR amplifications. The expected specific amplification products of *T. equi* and *B. caballi* were detected at 229 and 224 bp, respectively. Amplicons were purified for sequencing using the QIA quick Spin PCR Purification kit (Qiagen, Courtaboeuf, France). Sequencing of the PCR products was conducted by the GATC Company using an ABI 3730xl DNA sequencer. Each sequencing reaction was repeated three times in forward and reverse directions before being accepted for this study.

### Analysis of *rap-1* genes amplified from *B. caballi* - infected horses

Total gDNA was extracted of blood from infected horses as described above and PCR-amplified using primer set FS Rap-1 Forward: (5′-ATG AGG TGT TCT GCG AGT T-3′) and FS rap-1 Reverse (5′- GAT GGA CGT CAA AGG TG-3′) to amplify the full size *rap-1* gene. The thermocycling conditions used were: 95 °C for 3 min, followed by 35 cycles, consisting of denaturation at 95 °C for 30 s, annealing at 55 °C for 30 s and extension at 72 °C for 2 min. A final extension cycle at 72 °C for 7 min was performed and reactions were cooled to 15 °C. Three PCR amplicons were cloned into pCR®2.1-TOPO® (Life Technologies) and fully sequenced.

### Hematological investigations

A set of 48 blood samples were selected for hematological analysis according to the following criteria: (i) a control group of 12 samples from horses that are negative for infection to *B. caballi* and/or *T. equi*; (ii) a positive group of 12 samples from horses that were positive to blood film examination; (iii) and a positive control group with nine samples derived from horses which demonstrated positive cELISA reaction; (iv) nine samples derived from horses with nPCR positive results; and (v) a group of six samples derived from horses that were positive in all tests. All samples were evaluated using an automatic counter (MEDONIC CA620, Sweden). The parameters evaluated in the hemogram included erythrogram: red blood cell count (RBCs), hematocrit (HCT), hemoglobin (Hb) concentration and red cell indices; mean corpuscular volume (MCV), mean corpuscular hemoglobin (MCH), mean corpuscular hemoglobin concentration (MCHC), red blood cell distribution width absolute (RDWA), red cell distribution width percentage (RDW%), Leukogram: white blood cell count (WBCs), lymphocytes, granulocytes and mid cells (MID) and platelet count (PLT), mean platelet volume (MPV), platelet distribution width (PDW), plateletcrit (PCT), large platelet concentration ratio (LPCR).

### Statistical analysis

Chi-square (χ^2^) test was applied at probability of *P* < 0.05 to compare infection rates between equids, and to estimate the relative efficacy of the diagnostic methods for the detection of infected animals. Hematological data were subjected to statistical analysis including calculation of mean and standard error. Differences between groups were tested for significance using a one-way analysis of variance (ANOVA) followed by Duncan’s multiple range tests. Differences were considered significant at *P* < 0.05 level [23] using the SPSS version 14 software.

## Results

### Microscopic, serological, and molecular analysis of equine piroplasmosis

Direct microscopic examination of 139 equids samples detected 19 (13.6 %) positive samples [10 (11.4 %) positive horses and 9 (17.8 %) positive donkeys] (Table 2).

**Table 2:** Results of infected *T. equi* and/or *B. caballi* horses, donkeys and total equines detected by cELISA, nPCR, IFAT and blood film

	Horses (*N* = 88)								Donkeys (*N* = 51)								Equines (*N* = 139)							
	cELISA		nPCR		IFAT		Blood film		cELISA		nPCR		IFAT		Blood film		cELISA		nPCR		IFAT		Blood film	
	n	(%)	n	(%)	n	(%)	n	(%)	n	(%)	n	(%)	n	(%)	n	(%)	n	(%)	n	(%)	n	(%)	n	(%)
*T. equi*	13	14.8	32	36.4	21	23.9	–	–	12	23.5	22	43.1	16	31.4	–	–	25	18.0	54	38.8	37	26.6	–	–
*B. caballi*	0	0	17	19.3	15	17.0	–	–	0	0	8	15.7	16	31.4	–	–	0	0	25	18.0	31	22.3	–	–
EP	13	14.8	49	55.7	36	40.9	10	11.4	12	23.5	30	58.8	32	62.7	9	17.8	25	18.0	79	56.8	68	48.9	19	13.6

*N* number of animals tested, *n* number of infected animals, *%* percentage of infected animals

Testing of sera from the 139 equines by IFAT detected 21 (23.9 %) and 15 (17.0 %) out of 88 horses positive for *T. equi* and *B. caballi* respectively, and 16 (31.4 %) out of 51 donkeys tested positive for *T. equi* and 16 (31.4 %) for *B. caballi* (Tables 2 and 3). Therefore, 26.6 % of the combined equine serum samples tested contained antibodies reactive with *T. equi*, whereas 22.3 % of the equine serum samples contained antibodies reactive with *B. caballi* parasites as tested by IFAT (Table 2). Statistical analysis of these data using a χ^2^ test results shows no significant differences in the rate of infections with piroplasms between donkeys and horses using IFAT (Table 3).

**Table 3:** Results of cELISA, nPCR, IFAT and blood film for detection of *T. equi* and/or *B. caballi* in horses and donkeys

Animal	No. of tested animals	cELISA				nPCR				IFAT				Blood film	
		*T. equi*		*B. caballi*		*T. equi*		*B. caballi*		*T. equi*		*B. caballi*		*Equine piroplasmosis*	
		No. of positive	Infection (%)	No. of positive	Infection (%)	No. of positive	Infection (%)	No. of positive	Infection (%)	No. of positive	Infection (%)	No. of positive	Infection (%)	No. of positive	Infection (%)
Horses	88	13	14.8	0	0	32	36.4	17	19.3	21	23.9	15	17.0	10	11.4
Donkeys	51	12	23.5	0	0	22	43.1	8	15.7	16	31.4	16	31.4	9	17.8
EP	139	25	18.0	0	0	54	38.8	25	18	37	26.6	31	22.3	19	13.6
χ^2^		0.040		–		1.852		3.240		0.676		0.032		0.053	
Sig.		0.841		–		0.174		0.072		0.411		0.857		0.819	

Data was analyzed by χ^2^ and Sig. represents statistical significance for *P* < 0.05

Collectively, of the 88 horses and 51 donkeys tested by cELISA, 13 (14.8 %) and 12 (23.5 %) were positive for *T. equi*, respectively. Statistical analysis of these data using a χ^2^ test result showed, again, no significant differences in the rate of *T. equi* infections between donkeys and horses (Table 3).

Unexpectedly, and despite the identification of antibodies against *B. caballi* in 31 equine serum samples using IFAT (Tables 2 and 3), the *B. caballi* cELISA test failed to provide concordance for any positive samples when performed on all equid samples, despite the correct performance of the positive and negative controls provided by the cELISA manufacturer.

Nested-PCRs results to detect *T. equi* and *B. caballi* performed in all 139 equids sampled (88 horses and 51 donkeys) are shown in Tables 2 and 3. The prevalence of *T. equi* and *B. caballi* in equids were 38.8 % and 18 %, respectively. However, χ^2^ testing (Table 3) demonstrated no significant differences among *B. caballi* and *T. equi* infections in horses and donkeys using the nPCR data. Co-infection with both *T. equi* and *B. caballi* was found by nPCR in fifteen equids, 2 horses (2.3 %) and 13 donkeys (25 %).

**Fig. 1:**
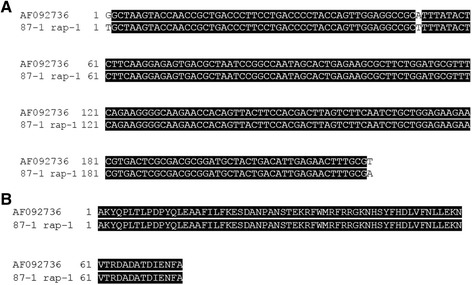
Alignments of the DNA **a** and predicted amino acid sequences **b** among Egyptian isolate termed 87-1 rap-1 (GenBank accession no. KR811096) and its equivalent region in the BC48 rap-1 reference gene sequence (GenBank accession no. AF092736)

Sequence analysis of two 229 bp nPCR amplicons, using *T. equi* internal specific primers (Table 1), demonstrated 100 % identity to the *B. equi* E12 ema-1 reference sequence (GenBank Accession number AF261824). In addition, sequencing of two 224 bp nPCR amplicon products of *B. caballi* obtained from Egyptian horses demonstrated identical sequences between them (GenBank accession number KJ094947). Comparison of the 224 bp amplicon and the predicted resulting amino acid sequences of the Bc48 Egyptian genotype sequences (87-1 rap-1) with the *B. caballi* clone X6 rap-1 reference sequence (GenBank Accession number AF092736), demonstrated three synonymous substitutions in this *rap-1* gene region of Egyptian genotypes (Fig. 1a, b).

### Comparative diagnostic performance of the cELISA, IFAT and nPCR tests used for surveillance of equine piroplasmosis

The diagnostic performances of the three methods used in the study were then statistically compared using a χ^2^ test (Table 4). The results confirmed that the nPCR technique detected significantly higher *T. equi* infection rates compared to cELISA in horses (*χ*^*2*^ = 10.65, *df* = 1, *P* < 0.001). However, no differences among the nPCR and IFAT tests were found for the detection of equine piroplasmosis, and no significant differences were found among the cELISA and IFAT tests for the detection of *T. equi* infections.

**Table 4:** Representation of statistical analysis comparing number of infected animals for *T. equi* and *B. caballi* by cELISA, IFAT and nPCR

Animal	cELISA *vs* nPCR		cELISA *vs* IFAT		nPCR *vs* IFAT			
	*T. equi*		*T. equi*		*T. equi*		*B. caballi*	
	χ^2^	Sig.	χ^2^	Sig.	χ^2^	Sig.	χ^2^	Sig.
Horses	8.022	0.005*	1.882	0.170	2.283	0.131	0.125	0.724
Donkeys	2.941	0.086	0.571	0.450	0.947	0.330	2.667	0.102
Total	10.646	0.001*	2.323	0.128	3.176	0.075	0.643	0.423

Data was analyzed by χ^2^ and Sig. represents statistical significance for *P < 0.05

### Sequence analysis of *rap-1* genes amplified by PCR in Egyptian *B. caballi* - infected equines

**Fig. 2:**
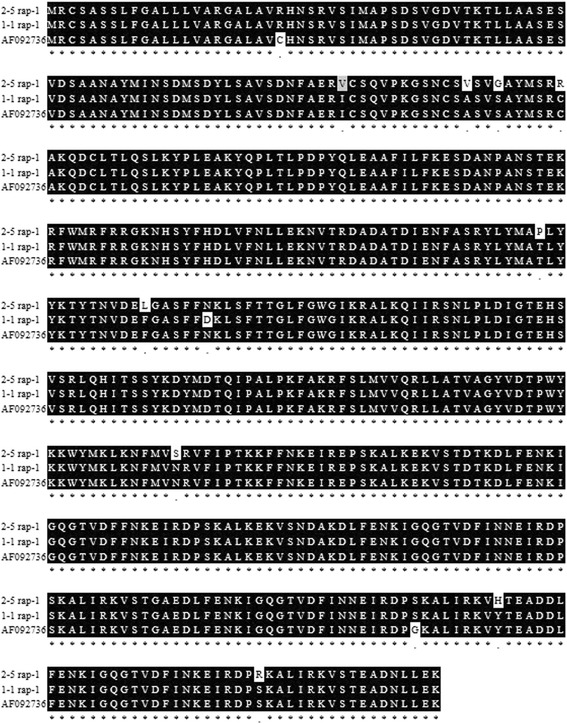
Alignments of the predicted amino acid sequences among the PCR amplicon derived from the reference gene sequence (GenBank accession no. AF092736), and two predicted RAP-1 amino acid sequences, termed 2-5 RAP-1 (GenBank accession number KR811097) and 1-1 RAP-1 (GenBank accession number KR811095), derived from Egyptian isolates of *B. caballi*

Sequence analysis of the nPCR amplicons and IFAT demonstrated the presence of *B. caballi* - infected equines in Egypt. While none of the horse or donkey samples tested in this study were demonstrated to be positive in the *B. caballi* RAP-1 based cELISA, comparisons among *rap-1* Egyptian sequences with the reference *rap-1* sequence shows high nucleotide conservation in a short portion of the full *B. caballi rap-1* gene. The failure of the *B. caballi* RAP-1 based cELISA assay (shown in Additional file 1: Table S1 and Additional file 2: Table S2) prompted further investigation of the full size *rap-1* gene sequences present in the Egyptian *B. caballi* isolates. nPCR using a set of primers designed to amplify the full size *B. caballi rap-1*gene (1943 bp) was performed on gDNA extracted from blood of three Egyptian *B. caballi* infected horses. Three distinct types of PCR amplicons termed “2-5 rap-1”, “1-1 rap-1”, and “87-3 rap-1” were initially generated using this approach. The *in silico* translation of the 87-3 *rap-1* gene (GenBank Accession number KR811096), demonstrates a truncated open reading frame (ORF) (Additional file 2: Table S2 and Additional file 3: Figure S1). In contrast, alignments of predicted amino acid sequences derived from sequences of the 2-5 rap-1 and 1-1 rap-1 amplicons (GenBank accession number KR811097 and KR811095 respectively) with the reference *B. caballi* RAP-1 sequence (GenBank accession number AF092736) demonstrated uninterrupted open reading frames in both genes encoding for a predicted 55 kDa rap-1 protein, containing a predicted signal peptide. (Fig. 2). A matrix representing the percentage of amino acid identities among these putative RAP-1 proteins is shown in Additional file 1: Table S1.

### Hematological findings

Results from the hematological analysis are shown in Table 5. Statistical analyses of the hematological results using one-way analysis of variance followed by Duncan’s multiple range tests and the χ^2^ revealed that the number of erythrocytes were markedly decreased  (ANOVA: *F*_(4.488)_, *P* = 0.004), while MCV values were significantly increased (ANOVA: *F*_(4.578)_, *P* = 0.004) in all EP positive equids*,* as detected by different techniques [blood film, cELISA, and nPCR testing or by all of them (blood film + ELISA + nPCR)] compared to the non-infected control group (12 horses that were negative for EP in blood film examination, cELISA, and nPCR). The Hb concentrations were significantly decreased (ANOVA: *F*_(2.813)_, *P* = 0.037) in infected groups detected by blood film, cELISA and nPCR techniques. However, there was a non-significant difference in HCT, MCHC and RDWA values in all EP positive equids compared to non-infected animals. This result indicated that EP potentially caused macrocytic hypochromic anemia in persistently infected horses (Table 5).

**Table 5:** Hematological profile in non-infected and infected horses with *T. equi* and/or *B. caballi*

Groups Parameters	Control Non-infected	Diagnostic techniques performed on infected horses				Sig.
		Blood film	cELISA	nPCR	Blood film + ELISA + PCR	
No. of Animals	12	12	9	9	6	
Red blood cell count (×10^6^/μl)	7.54 ± 0.19^a^	6.59 ± 0.23^b^	6.74 ± 0.30^b^	6.33 ± 0.13^b^	6.65 ± 0.31^b^	**
Haematocrit (%)	32.79 ± 0.86	31.14 ± 0.80	31.40 ± 1.52	29.01 ± 0.43	30.75 ± 2.22	NS
Haemoglobin (g/dl)	11.91 ± 0.27^a^	10.96 ± 0.26^ab^	11.20 ± 0.49^ab^	10.37 ± 0.12^b^	11.03 ± 0.66^ab^	*
Mean corpuscular volume (fl)	43.53 ± 0.70^b^	47.42 ± 0.53^a^	46.59 ± 0.77^a^	45.90 ± 0.58^a^	46.07 ± 1.47	**
Mean corpuscular haemoglobin (pg)	15.82 ± 0.18^b^	16.71 ± 0.25^a^	16.61 ± 0.12^a^	16.47 ± 0.29^ab^	16.57 ± 0.37^a^	*
MCHC (mg/dl)	36.40 ± 0.30	35.23 ± 0.29	35.73 ± 0.40	35.86 ± 0.24	36.08 ± 0.45	NS
Red blood cell distribution width (fl)	37.89 ± 1.08	40.96 ± 0.66	40.84 ± 1.04	41.04 ± 0.48	40.83 ± 2.35	NS
Red blood cell distribution width (%)	21.23 ± 0.48	20.13 ± 0.69	20.77 ± 0.27	21.72 ± 0.48	21.30 ± 0.83	NS
White blood cell count(×10^3^/μl)	7.47 ± 0.32	7.60 ± 0.13	8.00 ± 0.71	6.36 ± 0.36	7.63 ± 0.62	NS
Lymphocytes(×10^3^/μl)	1.65 ± 0.11	1.54 ± 0.10	1.63 ± 0.23	1.34 ± 0.14	1.95 ± 0.29	NS
Lymphocytes (%)	22.22 ± 1.36	20.32 ± 1.33	20.12 ± 1.35	22.06 ± 2.08	25.50 ± 2.53	NS
Granulocytes (×10^3^/μl)	5.19 ± 0.28^ab^	5.53 ± 0.16^a^	5.69 ± 0.45^a^	4.34 ± 0.32^b^	5.05 ± 0.42^ab^	*
Granulocytes (%)	69.38 ± 1.54	72.68 ± 1.62	72.04 ± 1.23	68.22 ± 2.55	66.80 ± 3.06	NS
Mid cells(×10^3^/μl)	0.63 ± 0.03	0.53 ± 0.03	0.68 ± 0.06	0.67 ± 0.09	0.63 ± 0.06	NS
Mid cells (%)	8.39 ± 0.36^ab^	7.00 ± 0.33^b^	7.83 ± 0.36^b^	9.72 ± 0.85^a^	7.70 ± 0.64^b^	**
Platelet count(×10^3^/μl)	220.58 ± 16.98^a^	136.17 ± 5.28^b^	140.22 ± 10.57^b^	139.67 ± 11.97^b^	152.67 ± 12.00^b^	***
Mean platelet volume (fl)	8.28 ± 0.25^a^	7.30 ± 0.21^b^	7.32 ± 0.17^b^	8.06 ± 0.15^ab^	7.77 ± 0.30^b^	**
Platelet distribution width (fl)	14.58 ± 0.34^a^	12.78 ± 0.49^c^	13.21 ± 0.25^bc^	14.20 ± 0.29^ab^	13.50 ± 0.53^abc^	**
Plateletcrit (%)	0.19 ± 0.02^a^	0.10 ± 0.01^b^	0.10 ± 0.01^b^	0.11 ± 0.01^b^	0.12 ± 0.01^b^	***
Large platelet concentration ratio	32.57 ± 2.45^a^	21.03 ± 2.33^c^	22.59 ± 1.16^bc^	28.42 ± 1.84^ab^	25.25 ± 3.28^bc^	**

Hemogram values expressed as mean ± standard error for each technique. Means followed by different superscripts (^a, b, c^) within the same row are significantly different at (*P* < 0.05). Two means that are not follow by the same letter (a, b, or c) are significantly different (*P* < 0.05). Two means followed with the same letter implies that they are not significantly different. Sig. represents statistical significance: *P < 0.05; **P <0.01; ***P <0.001; NS, non-significant

Statistical analysis performed on the leukogram data using the same statistical tools referred above showed that there was a marked (ANOVA: *F*_(2.664)_, *P* = 0.045) decrease in granulocyte counts (neutrophil + eosinophil + basophil) in EP positive horses. No significant difference occurred in WBCs, lymphocytes and MID (monocytes) counts in all EP positive equids in comparison with the non-infected control ones (Table 5).

Regarding the platelets; the PLT and PCT values revealed significant decrease (ANOVA: *F*_(9.743)_, *P* < 0.0001) and (ANOVA: *F*_(8.692)_, *P* < 0.0001), respectively, in all EP-positive equids compared to non-infected animals. Moreover, the MPV, PDW and LPCR values markedly decreased (ANOVA: *F*_(4.441)_, *P* = 0.004; *F*_(3.908)_, *P* = 0.009; and *F*_(4.835)_, *P* = 0.003) in piroplasma-infected animals detected by blood film and cELISA techniques compared to the other groups (Table 5).

## Discussion

Designing robust definitive studies on the incidence of EP in Egypt and globally, requires methods of proven efficacy. A goal of this study was to compare currently available EP diagnostic methods and to define their suitability for more definitive surveys in the country. Thus, the presence of *T. equi* and *B. caballi* infections in horses and donkeys in Egypt was evaluated using blood film, IFAT, nPCR, and cELISA methods [13, 24]. In addition, and to our knowledge, this study is the first report of the presence of *B. caballi* infections of equids in Egypt.

Microscopic examination of blood smears in the current study suggested lower EP incidence compared to what was reported before by Salib et al. [20] who found an incidence of 34 % for *T. equi* in horses using a similar microscopic examination method*,* and Farah et al. who reported incidences of 38.8 % for different horse populations in Egypt [19]. Comparison of microscopic examination data with the other techniques used in this study suggests that this method, although convenient due to its simplicity, likely, due to limited sensitivity, grossly underestimates the incidence of infection with parasites causing equine piroplasmosis.

In the present study, cELISA detected lesser number of *T. equi* infected animals than nPCR; however since only a small subset of nPCR products were sequenced, the accuracy of this comparison is unknown. However, based on the sequencing of the nPCR products and the consistent size of the amplicons produced in the reactions, it is reasonable to assume that nPCR would detect acute infections prior to serological assays. Consistently, the data in Table 4 shows statistically significant differences among the nPCR and cELISA for detection of *T. equi* infections in horses. These data are not unexpected since serological detection of infection with *T. equi* by cELISA requires four to six weeks post-infection [25]. The limited correlation found among these methods may be due to the fact that these two tests detect different entities (DNA *vs* antibodies) and thus differ in principle. Hence, while PCR can be considered reliable for diagnosis of active infection, serological tests are usually considered as the method of choice for detecting persistently infected animals [22, 24, 26].

The RAP-1 based cELISA demonstrated sensitivity for detecting antibodies against Venezuelan isolates of *B. caballi* [13] and for the detection of persistent-chronic infections [26]. Remarkably, the *B. caballi* cELISA test failed to detect any positives using equids sampled from Egypt. The cELISA data is not concordant with detection of *B. caballi* infection by IFAT and nPCR*,* since several horses and donkeys tested positive for *B. caballi* infection by these methods. Interestingly, a similar finding was previously reported in a study performed in South Africa, where no positive samples were identified using the same cELISA, and was attributed to extensive polymorphisms in the RAP-1 region containing the B-cell epitope defined by the mAb 79/17.18.5 used in the test [11]. In addition, a similar scenario was recently reported while analyzing the serological status to *B. caballi* in horses in Israel [12]. Sequence analysis on nPCR partial *rap-1* amplicons also demonstrated the presence of the Bc48/*rap-1* gene in Egyptian *B. caballi* strains. The portion of the Bc48 gene amplified and sequenced in this study using nPCR primers has a predicted amino acid sequence that is almost identical to the Bc48/*rap-1* gene of the Florida strain, used in the development of the mAb included in the *B. caballi* cELISA commercial kit used in this study [22]. Overall, the amplification of distinct full-sized *rap-1* sequences in each of the samples analyzed in this study suggests the presence of a diverse repertoire of *rap-1* genes in the Egyptian *B. caballi* isolates. Yet, while the South African and Israel *B. caballi* strains demonstrated dramatic *rap-1* polymorphisms including indel mutations [11, 12] when compared to the reference strain, the Egyptian strains showed a relatively limited degree of polymorphisms. However, whether the *rap-1* genes identified in this study are expressed during equid infections by the parasites, and if the observed sequence changes affect the immunogenicity of RAP-1 and/or the ability of the mAb 79/17.18.5 to bind the RAP-1 molecules from the Egyptian *B. caballi* isolates, remains unknown, and is beyond the scope of this study. Regardless of the reasons justifying the inability of the current *B. caballi* cELISA to detect infected equines in Egypt, these data suggest the need to improve serological diagnosis of *B. caballi* infection using alternative antigens and assays.

In this study, the most frequent blood alterations caused by EP include a reduction of the number of RBCs, PLT, HCT levels, Hb, MPV, PDW and LPCR, with increased values of MCV. While it cannot be disregarded that the blood alterations found in the samples analyzed may be due to other factors, collectively, these associations suggest that the piroplasma-infected horses suffered from macrocytic hypochromic anemia. Similar finding was recorded by Zobba et al. [27] in equine experimentally infected with piroplasms. Three mechanisms of hemolysis have been described: mechanical by trophozoite intra-erythrocyte binary fission [28], immune-mediated by auto-antibodies directed against components of the membranes of infected and uninfected erythrocytes, and toxicity by hemolytic factors produced by the parasite [29, 30]. There was regenerative anemia in infected horses associated with marked increase in RDWA due to anisocytosis and thrombocytopenia found in horses infected by *T. equi* and/or *B. caballi* has been rarely described in experimental and natural infections [31–33]. Although thrombocytopenia may be rarely reported in *T. equi* and/or *B. caballi* - infected horses, this is a very characteristic feature of canine babesiosis [31, 34]. The mechanism of thrombocytopenia in EP may be caused by local and systemic disseminated intravascular coagulation, immune-mediated destruction, and sequestration of platelets in the spleen [35]. Significant differences in total leukocyte counts and its differential between all groups of horses were found. Interestingly, taking together the data suggest numerous disturbances of the hemogram in chronically affected equines in the absence of acute signs of infections.

In summary, several currently used diagnostic tests were used in order to assess the presence of EP in a diverse equine population in Egypt. The overall incidence of infection with *T. equi* and/or *B. caballi* found in the population under study was relatively high regardless of the diagnostic method used (56.8 % using nPCR and 48.9 % using IFAT), and is in general agreement with previous assessments. Performance comparison of the IFAT and PCR tests used in this study shows no significant differences in their ability to detect infection of the causative agents of EP. Lack of concordance between the *B. caballi* cELISA, IFAT and nPCR suggests that a new diagnostic test based on additional antigen(s) will be needed. EP may be a contributing factor to hematological abnormalities of horses in Egypt.

## Conclusions

Data presented here support the use of IFAT, nPCR and cELISA for the detection of *T. equi* to assure identification of acute and chronic infection. Importantly, these assays represent key tools to design and implement more effective control measures. Absence of reactivity using serum samples from Egyptian equines in the *B. caballi* cELISA test indicates that improvements in the serological detection of *B. caballi* are needed. It is likely that EP is a cause for hematological abnormalities in horses in Egypt.

## Additional files

Additional file 1: Table S1. Percentage of the amino acid identity among reference gene (GenBank accession number AF092736) and the 2-5 and 1-1 RAP-1 full size Egyptian isolates (GenBank accession number KR811097 and KR811095). (DOCX 11 kb) Additional file 2: Table S2. Percentage of the DNA identity among reference gene (GenBank accession number AF092736) and the 2-5, 1-1 and 87-3 rap-1 full size Egyptian isolates (GenBank accession number KR811097, KR811095 and KR811096). (DOCX 11 kb) Additional file 3: Figure S1. Alignment of the DNA sequences among the reference gene (GenBank accession no. AF092736) and the 2-5, 1-1 and 87-3 *rap-1* full size Egyptian isolates (GenBank accession number KR811095, KR811096 and KR811097). A sequence gap is marked with a red box. (JPG 656 kb)

## Abbreviations

cELISA: competitive-enzyme-linked immune-sorbent assay
chi-square: χ^2^
EDTA: ethylene diamine tetra-acetic acid
ema: equi merozoite antigen
EP: equine piroplasmosis
fg: femptograms
gDNA: genomic DNA
Hb: hemoglobin
HCT: hematocrit
IFAT: indirect immunofluorescent antibody test
LPCR: large platelet concentration ratio
MCH: mean corpuscular hemoglobin
MCHC: mean corpuscular hemoglobin concentration
MCV: mean corpuscular volume
MID: mid cells
MPV: mean platelet volume
No.: number
nPCR: nested PCR
O.D.: optical density
ORF: open reading frame
PCR: polymerase chain reaction
PCT: plateletcrit
PDW: platelet distribution width
PLT: platelet count
rap-1: rhoptry associated protein-1
RBC: red blood cell count
RDW%: red cell distribution width percentage
RDWA: red blood cell distribution width absolute
WBC: white blood cell count
