# Supplementary material for: Assessment of Theileria equi and Babesia caballi infections in equine populations in Egypt by molecular, serological and hematological approaches
Source: Parasit Vectors. 2016 May 4;9:260. doi: 10.1186/s13071-016-1539-9 (PMC4857240; doi:10.1186/s13071-016-1539-9)
Supplement: Additional file 1: Table S1. — Percentage of the amino acid identity among reference gene (GenBank accession number AF092736) and the 2-5 and 1-1 RAP-1 full size Egyptian isolates (GenBank accession number KR811097 and KR811095). (DOCX 11 kb) [file 13071_2016_1539_MOESM1_ESM.docx]

**Supplementary Table 1A.** Percentage of the amino acid identity among reference gene (GenBank accession number AF092736) and the 2-5 and 1-1 RAP-1 full size Egyptian isolates (GenBank accession number KR811097 and KR811095).

| Sequence name | 2-5 rap-1 | 1-1 rap-1 | AF092736 |
| --- | --- | --- | --- |
| 2-5 rap-1 | 100.00 | 97.75 | 97.75 |
| 1-1 rap-1 | 97.75 | 100.00 | 99.39 |
| AF092736 | 97.75 | 99.39 | 100.00 |
