# Supplementary material for: Assessment of Theileria equi and Babesia caballi infections in equine populations in Egypt by molecular, serological and hematological approaches
Source: Parasit Vectors. 2016 May 4;9:260. doi: 10.1186/s13071-016-1539-9 (PMC4857240; doi:10.1186/s13071-016-1539-9)
Supplement: Additional file 2: Table S2. — Percentage of the DNA identity among reference gene (GenBank accession number AF092736) and the 2-5, 1-1 and 87-3 rap-1 full size Egyptian isolates (GenBank accession number KR811097, KR811095 and KR811096). (DOCX 11 kb) [file 13071_2016_1539_MOESM2_ESM.docx]

**Supplementary Table 1B.** Percentage of the DNA identity among reference gene (GenBank accession number AF092736) and the 2-5, 1-1 and 87-3 rap-1 full size Egyptian isolates (GenBank accession number KR811097, KR811095 and KR811096).

| Sequence name | 2-5 rap-1 | 1-1 rap-1 | AF092736 | 87-3 rap-1 |
| --- | --- | --- | --- | --- |
| 2-5 rap-1 | 100.00 | 99.11 | 99.11 | 87.76 |
| 1-1 rap-1 | 99.11 | 100.00 | 99.73 | 88.30 |
| AF092736 | 99.11 | 99.73 | 100.00 | 88.30 |
| 87-3 rap-1 | 87.76 | 88.30 | 88.30 | 100.00 |
