# Supplementary figures and images for: Assessment of Theileria equi and Babesia caballi infections in equine populations in Egypt by molecular, serological and hematological approaches
Source: Parasit Vectors. 2016 May 4;9:260. doi: 10.1186/s13071-016-1539-9 (PMC4857240; doi:10.1186/s13071-016-1539-9)

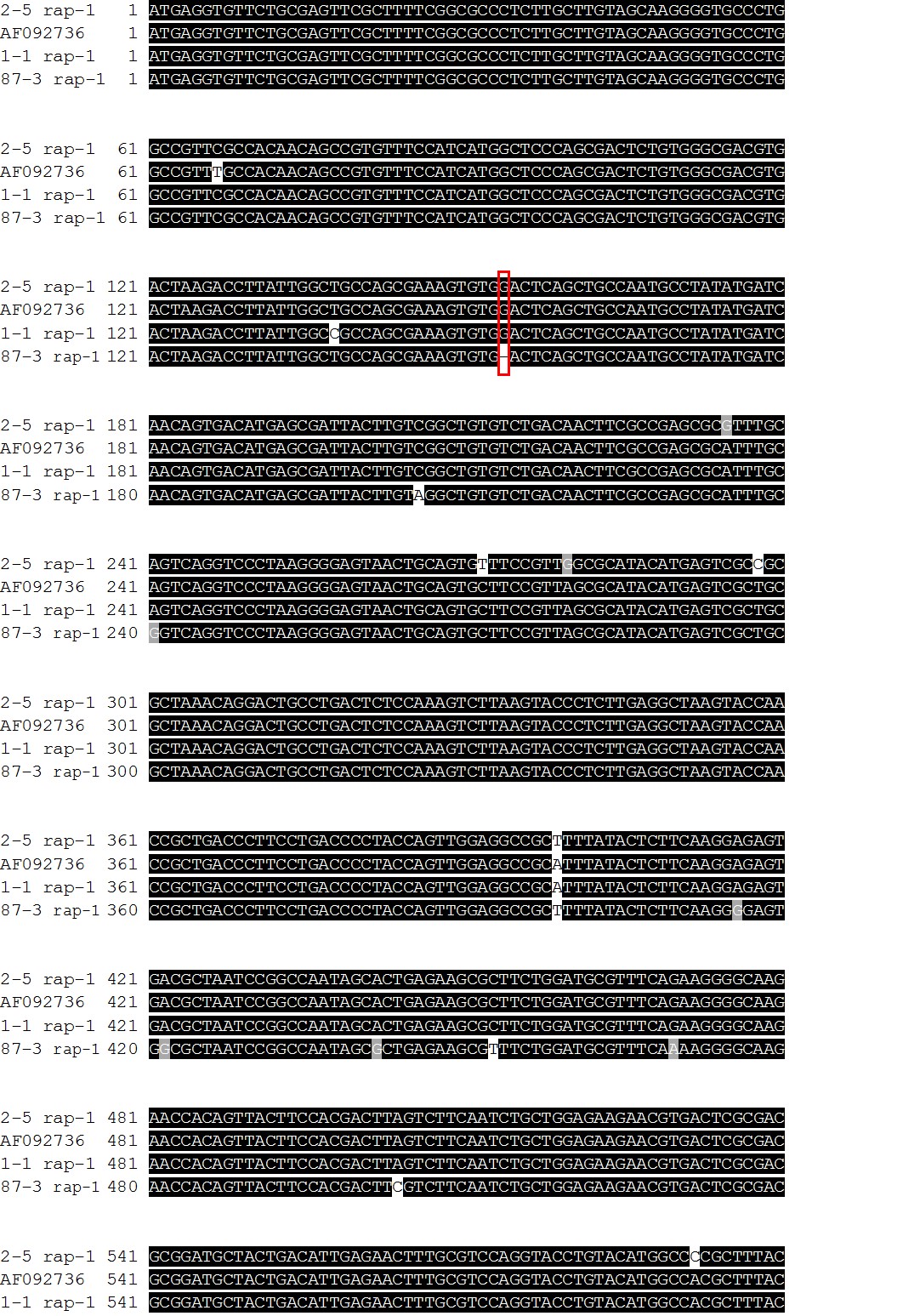

Supplement: Additional file 3: Figure S1. — Alignment of the DNA sequences among the reference gene (GenBank accession no. AF092736) and the 2-5, 1-1 and 87-3 rap-1 full size Egyptian isolates (GenBank accession number KR811095, KR811096 and KR811097). A sequence gap is marked with a red box. (JPG 656 kb) [file 13071_2016_1539_MOESM3_ESM.jpg]
